# Migraine is associated with altered approach–avoidance decision processing: a case control study

**DOI:** 10.1186/s10194-026-02445-5

**Published:** 2026-07-16

**Authors:** Sebastian Evers, Andrea Kübler, Claudia Sommer

**Affiliations:** 1 Department of Neurology, University Hospital Würzburg, Würzburg, Germany; 2 Research Training Group 2660 “Approach-Avoidance”, University of Würzburg, Würzburg, Germany; 3 Department of Psychology I, University of Würzburg, Würzburg, Germany; 4 Department of Anaesthesiology, Intensive Care, Emergency and Pain Medicine, University Hospital Würzburg, Würzburg, Germany

**Keywords:** High-density EEG, Migraine, Behavioral, Approach and avoidance, Trigger confrontation, Frontal-alpha-asymmetry, Frontal-midline-theta, Functional connectivity

## Abstract

**Background:**

Behavioral management of migraine has traditionally emphasized trigger avoidance, although emerging evidence supports a shift toward more confrontative coping strategies. Understanding how individuals with migraine process and respond to trigger-related information in approach-avoidance situations is critical for developing targeted interventions.

**Methods:**

Individuals with migraine and migraine-free controls were exposed to visual trigger-related and neutral stimuli prior to making approach-avoidance decisions in an adapted virtual T-maze paradigm. Behavioral measures included decision time (DT) and initial movement response (IMR). Neurophysiological activity was assessed through high-density EEG focusing on frontal alpha asymmetry (FAA), frontal midline theta activity (FMT), and functional connectivity as indexed by the debiased weighted phase lag index (dwPLI).

**Results:**

Both groups achieved comparable overall performance. Individuals with migraine exhibited significantly prolonged decision times (DTs), particularly in avoidance and high-conflict conditions, indicating increased cognitive demand. This effect was contrary to the initial hypothesis. Importantly, indirect trigger confrontation did not significantly influence avoidance behavior or decision times, nor did it modulate EEG measures, indicating a robust null effect. As secondary results, neurophysiological analyses revealed reduced FAA and FMT activity as well as situation-dependent alterations in dwPLI-based functional connectivity, providing converging evidence for altered regulatory control and increased neural excitability in migraine.

**Conclusions:**

Migraine is associated with altered approach–avoidance decision processing characterized by increased cognitive demand and impaired regulatory control. Indirect trigger confrontation appears insufficient to systematically modulate these processes within the present paradigm. These findings indicate alterations in decision-making dynamics in migraine. Further research using simpler behavioral paradigms and the utilization of neuromodulation strategies targeting FAA and FMT are required.

**Supplementary Information:**

The online version contains supplementary material available at 10.1186/s10194-026-02445-5.

## Background

With an estimated worldwide prevalence of around 14% [1, 2] and persistent lifelong symptoms that impact on the productive years of an individual [3], migraine is considered the most common and disabling headache disorder [4]. Typical migraine entails attacks of unilateral pulsating headaches with moderate to severe pain intensity, associated with nausea, vomiting, aggravation by routine physical activity as well as photo- and phonophobia [5]. Migraine is classified as either episodic or chronic depending on the monthly attack frequency. Over 70% of patients can identify individual precipitation factors, called “triggers”, that provoke a migraine attack [6, 7]. For a long time, behavioral therapy has focused on identification and avoidance of the triggers [6, 8]. Yet, studies have cast doubt on this approach.

From a learning-theoretical perspective, repeated avoidance may prevent habituation to trigger-related cues, potentially maintaining or even strengthening sensitivity over time. In line with this, Martin and colleagues proposed that reduced exposure to triggers may limit habituation processes, thereby increasing rather than decreasing reactivity to these cues in the long term [9]. Extending this view, Martin and McLeod argued that a strict “avoidance of triggers” philosophy should be replaced by a broader “coping with triggers” approach, which incorporates both avoidance and controlled exposure or confrontation strategies [10]. This conceptual shift aligns migraine trigger management with exposure-based principles used in other anxiety- and avoidance-related conditions.

Despite this theoretical development, relatively little is known about how individuals with migraine make behavioral decisions when confronted with migraine-associated cues, and which neurophysiological mechanisms underlie approach versus avoidance behavior in this context. In particular, it remains unclear how trigger-related decision making differs between individuals with and without migraine at both the behavioral and neural level. Migraine-related cues can acquire aversive motivational value through prior experience, leading to a potential bias toward avoidance when such cues are encountered. In an approach–avoidance context, however, participants may need to balance this avoidance tendency against competing approach motivations. This conflict may be reflected in behavioral choices that either favor withdrawal from or engagement with trigger-related stimuli. At the neural level, these processes are assumed to be supported by partially distinct but ictal acting systems. Frontal alpha asymmetry (FAA) has been linked to motivational direction, with relatively greater right-hemispheric activity associated with withdrawal-related behavior [11]. Midfrontal theta activity (FMT) has been implicated in cognitive control and conflict monitoring during decision making [12]. In addition, functional connectivity between brain regions is of interest, as previous studies have demonstrated altered connectivity patterns in migraine, including between occipital and frontal regions [13]. The present paradigm operationalizes this framework by requiring approach–avoidance decisions in response to migraine-related cues. This allows for an integrated investigation of migraine-related decision-making across behavioral and neural levels. The objectives of this study were (I) to validate an approach and avoidance paradigm for migraine trigger confrontation, (II) to compare the behavioral response of people with and without migraine, and (III) to explore potential differences in the neurophysiological correlates of approach and avoidance decision making, specifically in FAA, FMT and brain connectivity. We hypothesized that confrontation with migraine-related stimuli would elicit stronger avoidance behavior in individuals with migraine as compared to control participants, reflected in faster avoidance responses. On the neural level, we expected greater right-lateralized FAA, increased midfrontal theta activity as an index of conflict processing, and altered functional connectivity patterns, particularly within frontal networks. The answers to these hypotheses may set the foundation for capturing behavioral changes in people with migraine and open the door for potential treatments and interventions.

## Materials and methods

This study was designed as a prospective case-control study including people with migraine as a migraine group and an age- and gender-matched migraine-free control group. Participants underwent behavioral testing with simultaneous EEG recording. No interventions were applied. The study protocol was carried out in accordance with the Declaration of Helsinki (2024) and was approved by the Ethical Review Board of the Institute of Psychology of the Julius-Maximilians-University of Würzburg, Germany (GZEK 2023-07). The study was preregistered at the German Clinical Trials Register (DRKS00032084; registration date: 20 June 2023). Clinical trial number: not applicable.

### Recruitment

A sample size calculation of two groups with independent means was conducted with G*Power [14] with 𝛼 = 0.05, power(1-ß) = 0.8 and a large effect size of d = 0.8. This choice was based on theoretical and methodological considerations. The present case-control design compares individuals with migraine and migraine-free control subjects and is generally associated with medium-to-large behavioral and neurophysiological effects. Individually relevant trigger-related stimuli are expected to elicit strong responses from the migraine group due to their high motivational influence. Corresponding neurophysiological markers, including FAA and FMT-activity, have been shown to produce robust effects in paradigms assessing approach-avoidance motivation and cognitive control [15, 16]. Given the limited prior evidence in the current context and the partially exploratory nature of the outcomes, a large effect size was considered appropriate to detect clinically meaningful differences within a feasible study design. Accordingly, the necessary group size was 26 participants. For compensation of post-recording exclusion and age-matching purposes, we aimed at *n* = 30 participants per group. Participants in the migraine group self-reported their prior received clinical migraine diagnosis, which was subsequently screened for consistency with ICHD-3 criteria [17], including episodic and chronic migraine with and without aura. Inclusion criteria were age 18–70 years, normal hearing, and normal or corrected vision. Exclusion criteria included neurological or psychiatric disorders and use of psychoactive medication. Controls were age- and gender-matched and met the same criteria except for migraine diagnosis. Recruitment took place over an intranet announcement to the staff of the University Hospital, and through an internal study register of the Institute of Psychology, University of Würzburg. Prior to participation, both groups received information about the procedure, paradigm, goal and possible risks of this study and gave written informed consent. Participants received €22 plus a performance-based bonus of up to €5.

### Paradigm

#### Trigger confrontation

A pool of 54 migraine-related German words – taken from previous migraine questionnaire results and personal interviews – was reduced via an online survey (*n* = 117 migraine patients) to 24 highly associated trigger words across four categories (Table 1A, grey fields). Each participant selected their top three individually relevant words (migraine group) or headache-related words (control group).

Control stimuli consisted of neutral and negative (non-headache-related) words and were randomly chosen for each participant. For the imaginative task, participants associated three symbols with personal memories (Table 1B) by briefly describing them in writing and recalling them during the presentation.

**Table 1:**
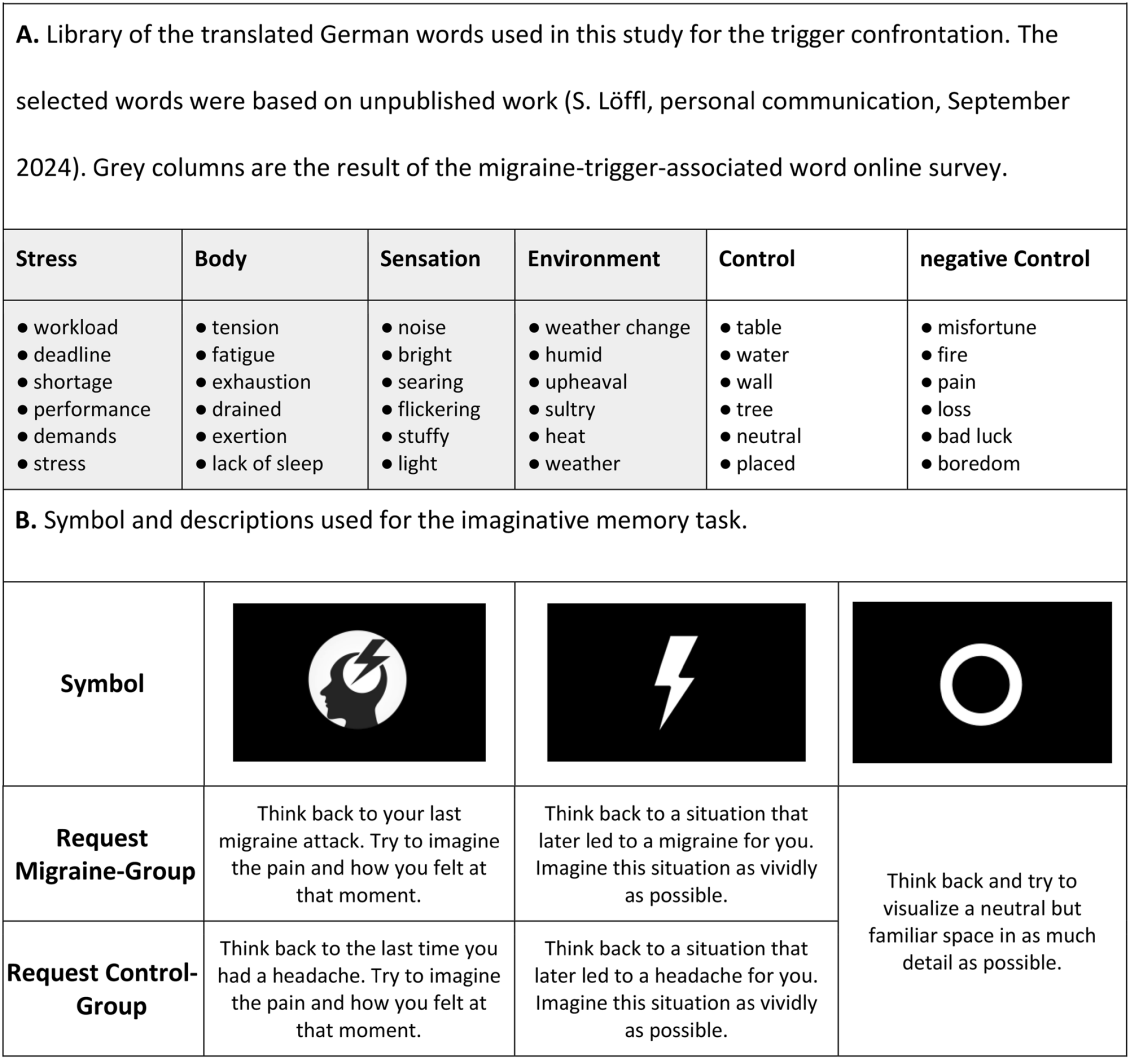
Visual trigger confrontation library (original German translation is shown in Additional file 1)

#### Behavioral T-maze

**Fig. 1:**
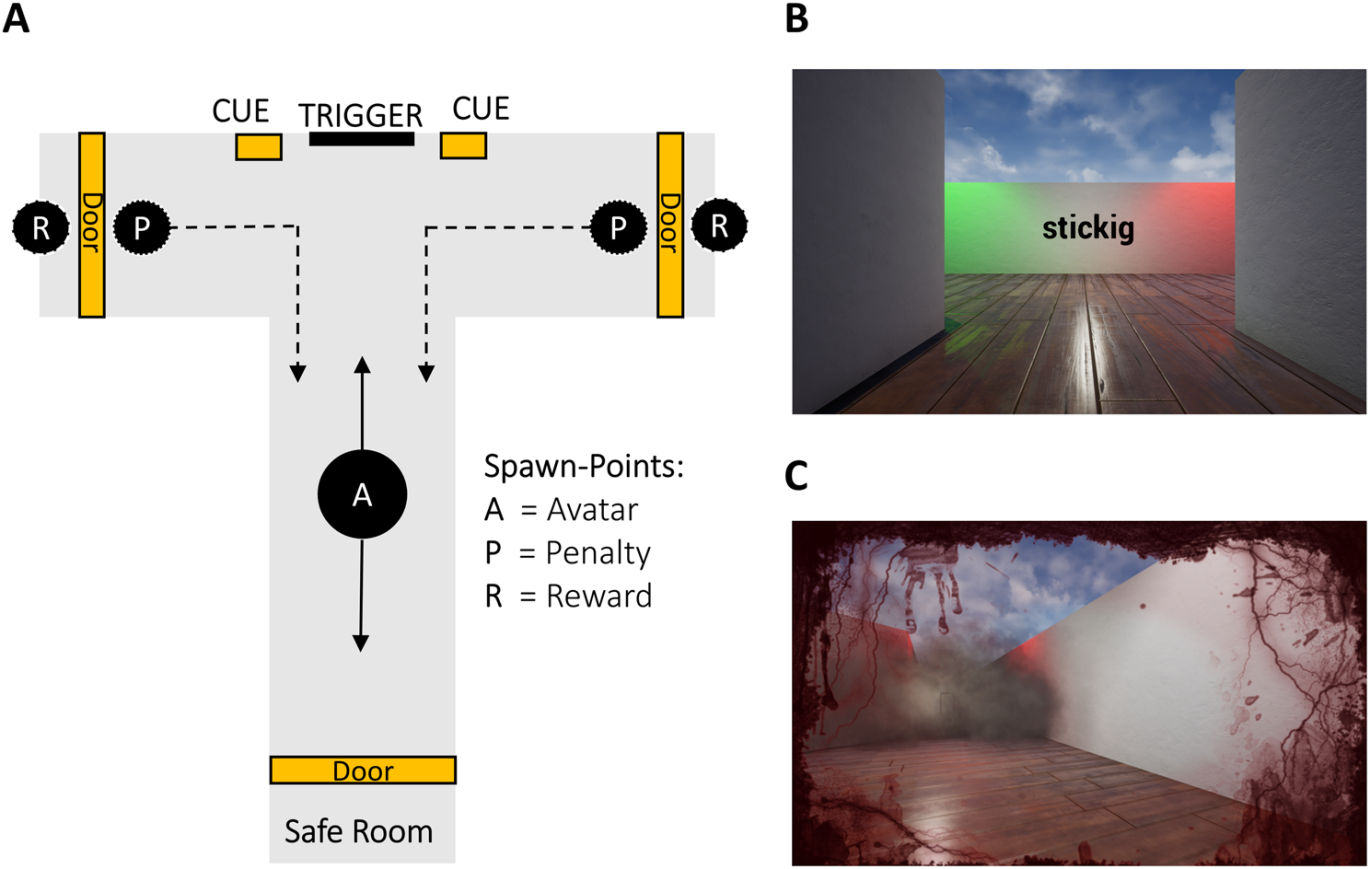
T-maze-paradigm design. **(A)** T-maze-blueprint **(B)** In-game representation with cues and confrontation (here “stickig”: German for stuffy) **(C)** Penalty-representation in close proximity

An adapted virtual T-maze paradigm was used to assess approach–avoidance behavior [18] (Fig. 1). Participants controlled an avatar with a handheld controller with the aim to collect points. The participant could reach either a reward room through an open door (+ 10), get caught by a pursuing penalty in the form of a creeping fog (Fig. 1C) (-10), retreat to a safe room (+ 1), or fail to reach one of the previous endpoints in time (0). It was not possible to reach more than one endpoint in one trial.

Trial conditions were indicated with a colored cue light coming from the corresponding side arm of the T-maze (Fig. 1B) and included:

(i) Approach (reward only, green light),
(ii) Avoidance (penalty only, red light),
(iii) Conflict (reward and penalty, red and green light), and
(iv) Ambiguity (uncertain outcome, yellow light).

Task parameters (e.g., penalty speed, movement constraints) were varied to maintain decision conflict while ensuring task engagement.

### Procedure

#### Study protocol

Participants attended a single session. After consent, they completed questionnaires assessing migraine characteristics (ICHD-3) [5], demographics, and headache trigger sensitivity (HTSAQ-SF) [19]. Participants were seated in front of a 24” monitor (50–60 cm distance) and equipped with the EEG recording device. Individual trigger words were selected, and the imaginative task was prepared. Prior to the actual T-maze task, the participants were familiarized with the controls and the task via a tutorial. The T-maze task itself consisted of 120 trials (5 confrontation categories × 3 stimuli × 4 conditions × 2 repetitions), presented in blocks of 30 trials with self-paced breaks. After all trials were finished, participants were asked about their experiences and impressions of their performance in this study. Participants were instructed not to attend the experiment if experiencing headache symptoms, and were monitored during testing for the onset of migraine symptoms, with the instruction to terminate the session if necessary. Information regarding proximity to migraine attacks was obtained after completion of the experiment from patients’ migraine diaries.

#### Single trial structure

Each trial began with a fixation period (2.5 s), followed by trigger confrontation (6 s for words, 10 s for imagery). The screen then faded to the T-maze task (2.5 s) with the confrontation stimuli displayed in black on the front-facing wall of the maze. After 1 s, the cue of the trial condition was shown and with it the possibility to virtually move. The program marked this timepoint as the cue-point for later analysis and recorded the virtual movement as well as the thumbstick input values of the controller. The participants had up to 10 s to reach one of the possible endpoints.

**Fig. 2:**
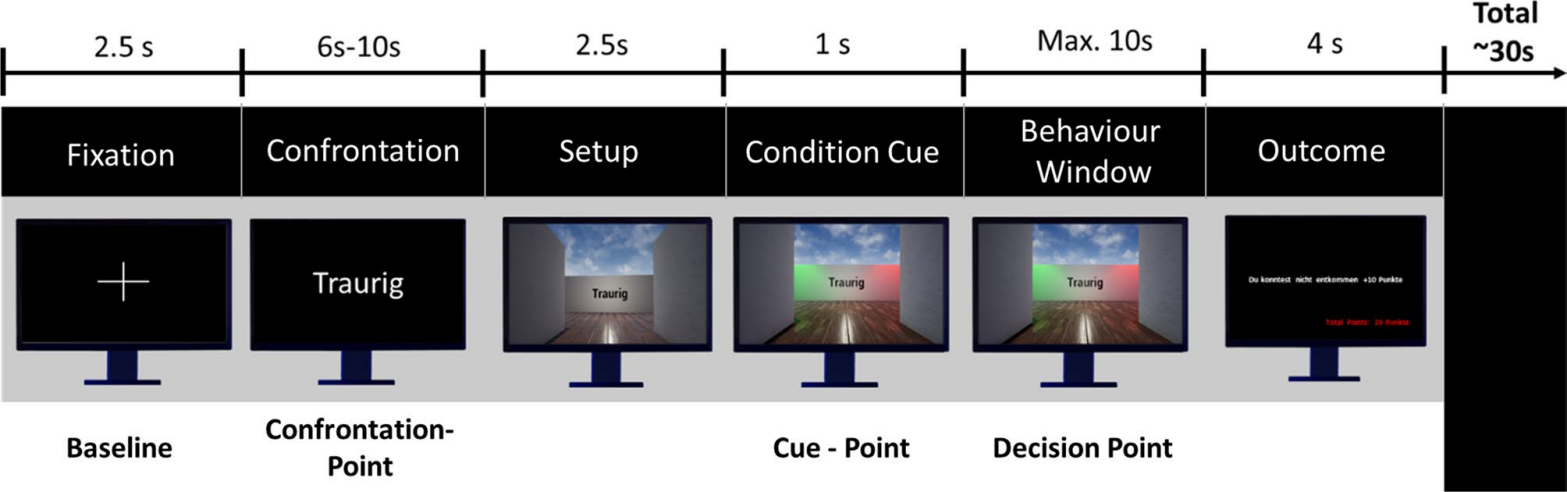
Timeline of a single trial. (Traurig: German for sad)

As soon as one endpoint was reached, the trial ended and the trial outcomes were displayed (4 s) resulting in a total trial duration of 20–30 s. The complete single trial structure is shown in Fig. 2.

#### Recording setup

The setup of the study consisted of a controlling computer and a recording laptop that were both connected through a local hub in a private network. The controlling computer was connected to two monitors, one for the participant in the testing room, and one for the supervisor. Two loudspeakers for the auditory feedback, a controller for the participant input, as well as the EEG-amplifier were connected to this computer. The paradigm and trigger confrontation were operated in the Unreal Engine [20] and were synchronized with the EEG-amplifier and the recording laptop via a Python script and LabStreamingLayer (LSL) [21].

#### EEG-recording

EEG was recorded with a passive 128 channel “Brain Product R-Net” (Brain Products GmbH, Gilching, Germany) with passive semi-dry Ag/AgCl electrodes at a sampling frequency of 500 Hz.

The EEG arrangement followed the standard 128 channel buildup in accordance with the extended version of the international 10–20 system with an included ground (Fpz) and reference electrode (FCz). To allow recording of all electrodes, four 32-channel “BrainAmp”-EEG amplifiers were used and connected to the study computer. Based on the semi-dry system requirements of the manufacturer, impedances were kept below 20 kΩ. The data were transmitted continuously by the study computer into the local network, through the “LSL-BrainAmpSeries” Software and then saved with the LSL LabRecorder on the recording computer. In case the participants were wearing glasses, the electrodes F9, F10, FT10 and FT9 were removed to ensure that they could participate at optimal visual performance.

For the present analyses, EEG electrodes were defined a priori for region-of-interest (ROI) analyses based on established neurophysiological evidence on frontal alpha asymmetry (FAA), midfrontal theta activity (FMT), and previously reported migraine-related connectivity alterations. Two frontal clusters in each hemisphere were defined for FAA, and a central midfrontal cluster for FMT.

For connectivity analyses, additional bilateral electrode clusters were defined in temporal, parietal, and occipital regions to provide comprehensive and symmetric scalp coverage within a balanced network framework. These clusters were constructed to ensure comparable cluster sizes across all regions, enabling consistent network-level comparisons rather than being based on region-specific prior hypotheses. A small number of electrodes were not included in any cluster due to this parcellation approach.

**Fig. 3:**
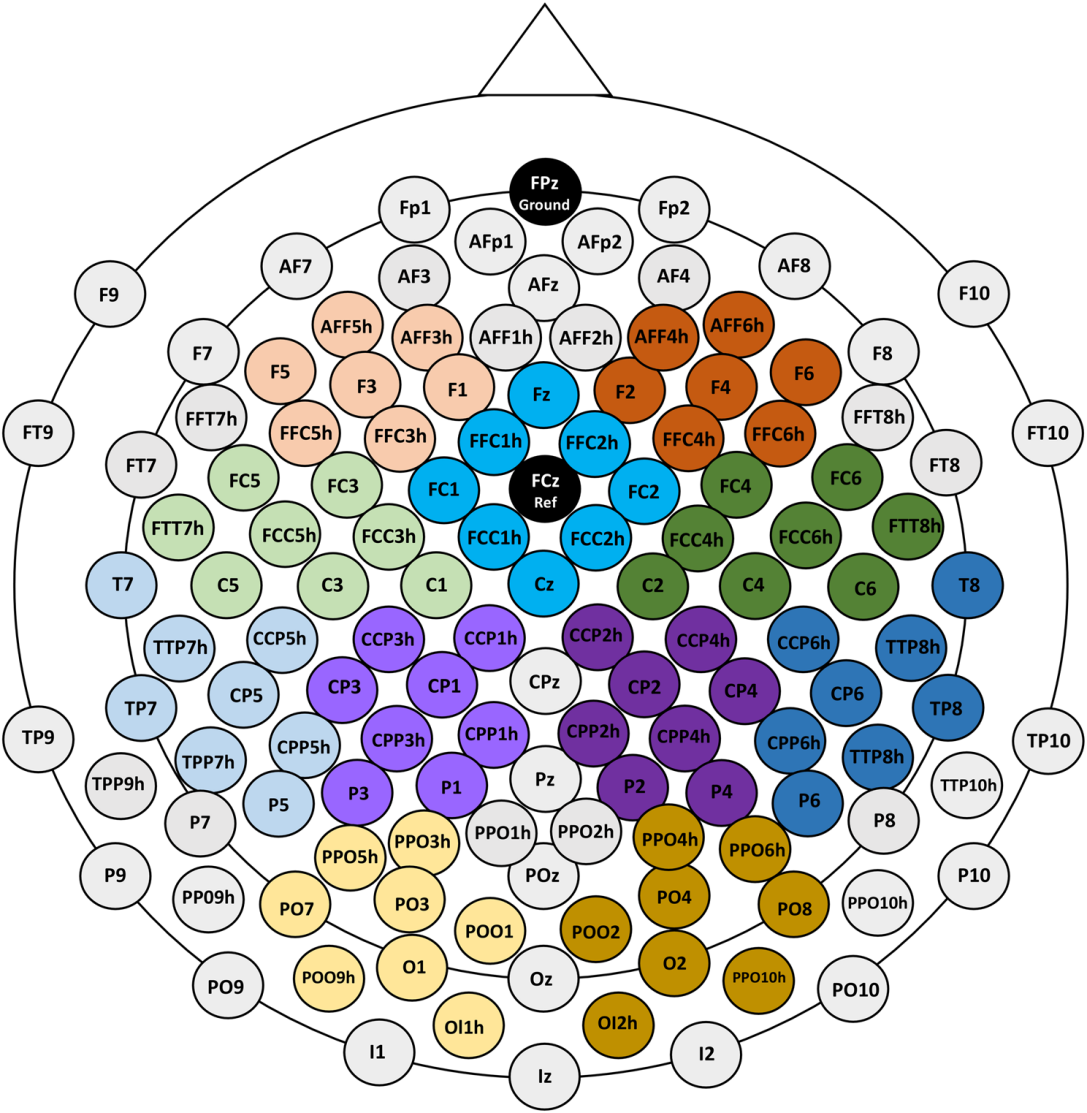
R-Net (BrainVision) 128 channel layout. For the analysis the channels were structured into 11 clusters and here marked with different colors. The lighter colors encode the left hemisphere, while darker colors mark the right hemisphere. Orange=Frontal Cluster, Green: Central Cluster, Blue= Temporal Cluster, Purple=Parietal Cluster, yellow=occipital cluster. The exception is the midfrontal cluster in bright blue which is single and has no counterpart

Overall, this procedure resulted in 11 clusters: five per hemisphere (frontal, temporal, central/parietal, and occipital regions) and one midline cluster (midfrontal) (Fig. 3).

### Pre-processing

#### Behavioral measures

Decision time (DT) was defined as the first sustained controller input change within 4 s after cue onset, exceeding 20% deviation for at least 400 ms. Trials without valid DTs or with DTs exceeding 4 s were excluded (2%).

Initial movement response (IMR) was calculated based on movement trajectories calculated by the position development (recorded in 30 Hz) within the first 4 s of the task, weighted toward early movement using a logarithmic scaling function. The IMR could reach values between − 1200 and + 1200. The viewing angle of the avatar was not taken into consideration.

#### EEG

Raw EEG data were transformed into BIDS-format and then preprocessed with the MNE-toolbox in the “anaconda” python environment. The raw stream was high-pass filtered at 0.1 Hz with a zero-phase 4th order (FIR) filter. To ensure a conservative quality-control approach without unnecessarily rejecting valid channels, a threshold of 30 kΩ was used to identify faulty electrodes for interpolation.

Mean impedance was 16.1 ± 9.72 kΩ (control) and 20.7 ± 11.5 kΩ (migraine), showing a significant group difference (*p* = 0.001) but no evidence of systematic spatial clustering across the scalp. Overall, 7.26% of channels were interpolated (control: 6.1%, migraine: 8.6%).

The signal was then re-referenced to the common average reference (CAR) and low-pass filtered at 30 Hz with a 4th order zero-phase (FIR) filter. Independent component analysis (ICA) of the MNE-icalabel library [22] was used to automatically identify and remove body and eye-related artefacts (blinks and horizontal eye movements, heartbeats, etc.). Subsequently, each trial was split into 3 different epochs with a length of 2 s. The epochs were time-locked to the start of fixation, confrontation and condition cue of a single trial, respectively. The length of 2 s was chosen to include the higher brain processes involved in decision making (e.g., FAA at 8–13 Hz and FMT at 4–8 Hz) and to incorporate most DT-windows. Noisy epochs were rejected based on a peak-to-peak amplitude threshold of 300 µV, resulting in a mean rejection of 3% of the trials.

### Frequency domain analysis

The epochs were averaged per trial condition, followed by a fast Fourier transformation after Welch [23] in a range of 1–30 Hz. The calculated power of the confrontation and cue epochs were dB-baselined for the graphical plots and for the FMT but not for the FAA. Of this final result the theta and alpha bands were extracted and used to calculate the final scores of the FAA and FMT. The FAA was calculated as the commonly used right frontal alpha asymmetry (r-FAA) score [24]. Yet, to account for possible imprecisions in the placement of the EEG-cap and our high-density approach, we chose to select the mean of the designated frontal clusters (Fig. 3, bright and dark orange) for the calculations, instead of the typical single channel approach. The score was calculated by subtracting the logarithmic values of the mean of the left from right cluster. For the FMT, the mean of the designated midfrontal cluster of this study was used (Fig. 3, bright blue).

### EEG - connectivity analysis

Functional connectivity was estimated using the debiased Weighted Phase Lag (dwPLI) [25], which examines the consistency of time-delayed (non-zero-lag) oscillations while minimizing the influence of volume conduction and distortions caused by finite sample sizes.

The value of dwPLI can range from 0 to 1, with 0 representing no consistency and 1 representing a very stable non-zero-phase coupling whose phase remains consistent over time.

For each confrontation- and cue-point epoch, dwPLI was first calculated between all possible channel pairs across two clusters using the spectral_connectivity function of the MNE-connectivity library. Subsequently, all pairwise dwPLI values between the respective cluster combinations were averaged to obtain a single inter-cluster connectivity value. Thus, connectivity was estimated at the electrode-pair level prior to aggregation across cluster-links.

### Statistical analysis

All statistical analysis was conducted in R and its environment (v. 4.5.2) [26]. Group differences in electrode impedances were assessed using an independent-samples t-test.

Linear mixed-effects models (lme4) [27] were used to assess group differences, including fixed effects of group, condition, and confrontation, as well as their interactions, and trial number as a covariate. Random intercepts and slopes were included per participant. In some cases, additional fixed effects were added. For example, for the functional connectivity analysis, an additional between–subject factor representing cluster connections was added to the model.

P-values were obtained using Satterthwaite approximation (lmerTest) [28]. Post-hoc comparisons were conducted using estimated marginal means (emmeans) [29]. Tukey adjustment was applied for pairwise post-hoc comparisons within the behavioral and EEG models. For the functional connectivity analyses, false discovery rate (FDR) correction was applied to post-hoc comparisons involving multiple cluster-link levels to control for the increased risk of false-positive findings due to the large number of connectivity-related comparisons.

Outliers (> 3 SD within participant, trial condition and confrontation) were removed, and DT was log-transformed. Model assumptions were evaluated visually (performance) [30]. Statistical significance was set at α = 0.05. For all statistical results in this paper, we use ‘M’ to refer to the migraine group and ‘C’ to refer to the migraine free control group.

## Results

### Study population

A total of *N* = 32 people with migraine for the migraine group and *N* = 32 migraine-free people for the control group were enrolled. In the migraine group, 4 participants had to be excluded. Two were unable to complete the recording due to an emerging migraine attack and two others due to the intake of anti-depressants (amitriptyline, venlafaxine) that could possibly influence the EEG recording and general behavior. As a result, *n* = 28 people with migraine (mean age, 38.7 ± 13 years; 23 female) and *n* = 32 headache-free control subjects (mean age, 33.8 ± 13.9 years; 22 female) were analyzed. Out of all participants, 16 (57.1%) were strictly interictal, while 12 (42.8%) were tested within three days before or after a migraine attack (Additional File 2). The characteristics of the study sample are summarized in Table 2. Participants who reported a previous diagnosis of chronic migraine and a recent reduction in headache frequency under treatment were still classified as “chronic migraine”. These participants were retained in the chronic migraine group for exploratory subgroup analyses, as the reduced attack frequency was considered to reflect treatment-related symptom improvement rather than a change in migraine subtype. There was no significant difference in age (*t*(58) = -1.46, *p* = 0.15) or sex (*X*^2^ (1, *N* = 58) = 0.133, *p* = 0.715) between the groups. Both groups had a similar familiarity and experience with a handheld gaming controller (rating from 0 to 10, M = 4.5 ± 2.9, C = 5.25 ± 2.8; *t*(58) = 0.74, *p* = 0.467), and with a first-person-view gaming environment (M = 3.4 ± 2.9, C = 4.2 ± 2.9; *t*(52) = 0.826, *p* = 0.412). Both experience variables are strongly positively correlated (Pearson’s *r* = 0.72, *p* < 0.01, *n* = 60) and will be represented by the first-person-view gaming environment value (env_exp_val) in this paper. A summary of the study population can be seen in Additional file 3.

**Table 2:** Demographic and clinical characteristics of the migraine group

variable	Migraine group (*n* = 28)
Age, years	38.7 ± 12.9
Female sex, n (%)	23 (82.1)
Disease duration, years	19.5 ± 13.6
**Monthly migraine frequency**,** n (%)**	
• ≤4 attacks/month	7 (25.0)
• 5–8 attacks/month	10 (35.7)
• ≥15 attacks/month	11 (39.3)
**Self-reported Migraine subtype**,** n (%)**	
• Episodic migraine	12 (42.9)
• Chronic migraine^1^	16 (57.1)
**Aura status**,** n (%)**	
• Migraine with aura	16 (57.1)
• Migraine without aura	11 (39.3)
• not stated	1 (3.6)
**Medication status**,** n (%)**	
• Acute treatment only	18 (64.3)
• Preventive treatment ± acute medication	9 (32.1)
• No medication	1 (3.6)
HTSAQ trigger sensitivity	46.5 ± 9.6
HTSAQ avoidance	42.9 ± 9.8

^1^Among these, 5 reported a recent reduction in migraine frequency under medication

### Decision time (DT)

Overall decision times (DTs) were longer in the migraine group (M = 1.09 ± 0.64 s) as compared to the control group (C = 0.92 ± 0.43 s). To assess the effects of group, condition and confrontation on DT, a LMM was fitted including “env_exp_val” as a covariate to account for individual differences in prior environmental experiences. Descriptive means are summarized in Table 3, and a complete overview of all statistical and significance calculations are provided [see Additional file 4].

**Table 3:**
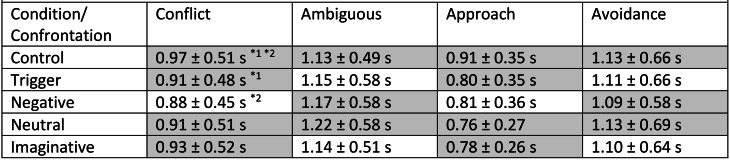
Grand average decision time (DT) across groups, for each combination of condition and confrontation

Grey shading denotes conditions that are significantly different (p< 0.05) from all other conditions within the same confrontation (row-wise comparison). Values marked with * are significantly different compared to the other marked value (column-wise comparison)

Grey shading denotes conditions that are significantly different (*p* < 0.05) from all other conditions within the same confrontation (row-wise comparison). Values marked with * are significantly different compared to the other marked value (column-wise comparison).

**Fig. 4:**
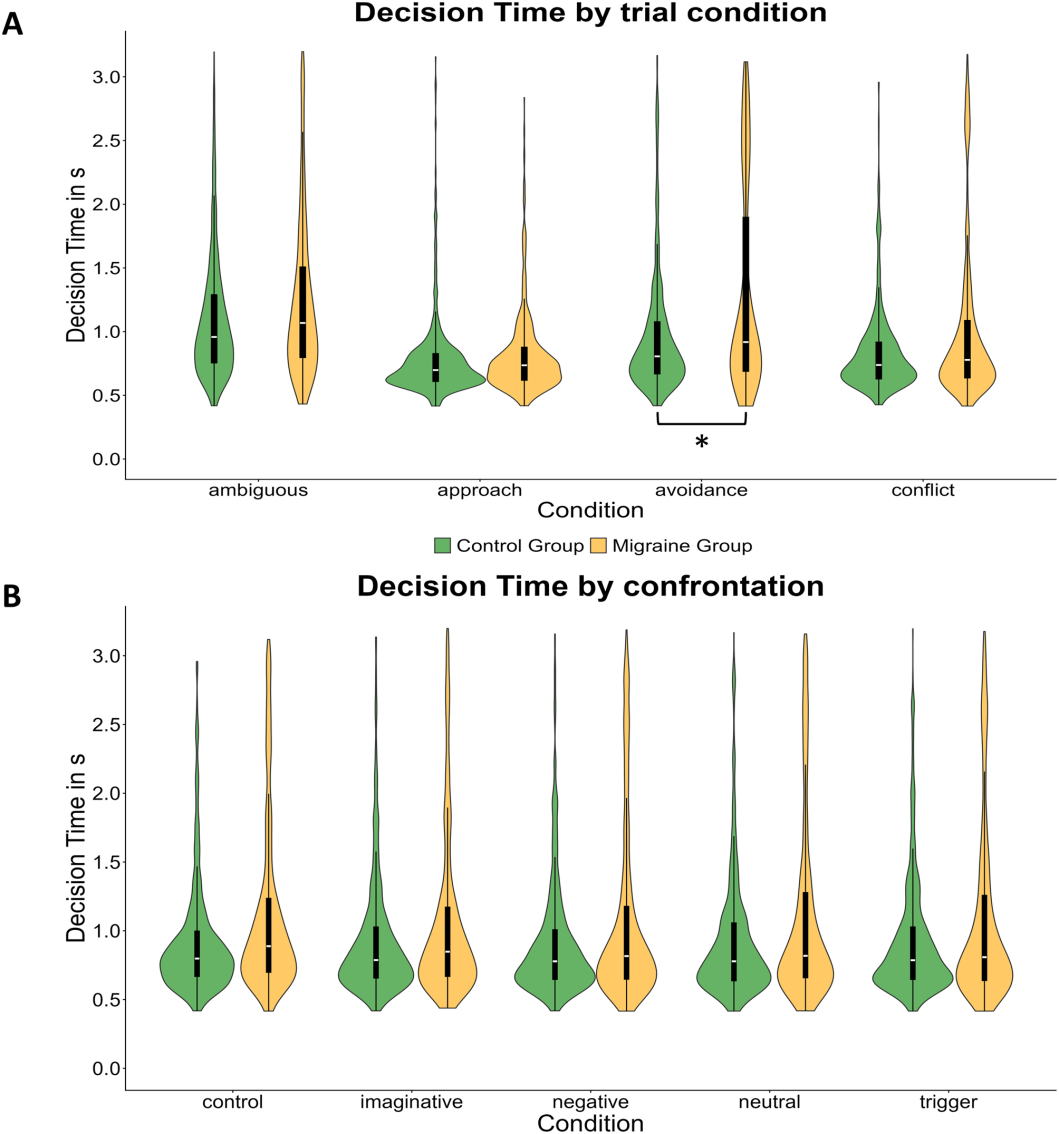
Recorded averaged decision times (DTs [s]) over categories and groups. **A**. DTs averaged over T-maze condition categories. **B**. DTs averaged over confrontation stimuli categories. Migraine = orange, Control = green, * = *p* < 0.05

The model revealed no significant three-way interaction. Instead, the group x condition interaction was found to be significant (*F*_(3, 58.1)_ = 3.26, *p* = 0.027; Fig. 4A), indicating that group differences in DT depended on trial condition. Post-hoc analyses showed significantly longer DT in the migraine group during avoidance trials (β = 0.23 ± 0.08 s, *t*(58) = 2.8, *p* = 0.007). DTs were also numerically longer in conflict (β = 0.11 ± 0.06 s, *t*(57.7) = 1.8, *p* = 0.078) and ambiguous trials (β = 0.12 ± 0.06 s, *t*(58) = 1.8, *p* = 0.075), although these differences only trended toward statistical significance. No group differences were observed in approach trials (β = 0.025 ± 0.04 s, *t*(57.2) = 0.58, *p =* 0.566). The interaction between condition x confrontation was also significant (*F*_(12, 6121.1)_ = 2.11, *p* = 0.016). Post hoc analyses revealed significant differences in DT between trial conditions for most condition–confrontation combinations [see Additional file 4]. However, some confrontation categories elicited similar DT across specific conditions. Specifically, trigger and imaginative confrontation produced comparable DT in ambiguous and avoidance conditions, whereas negative confrontation elicited similar DT in conflict and approach conditions. Overall, DTs were longest in ambiguous and avoidance combinations and shortest in the approach condition, although the relative ordering of conditions is still dependent on the confrontation type.

The group × confrontation interaction also approached significance (F_(4, 6120.9)_ = 2.27, *p* = 0.059), indicating that the effect of confrontation on DTs was similar across groups.

Aside from the interactions, a significant main effect of group was revealed with longer DT in the migraine group (F_(1, 56.6)_ = 5.81, *p* = 0.019, β = 0.119 ± 0.049 s). Significance was also observed for the main effect of trial condition (*F*_(3, 58.1)_ = 66.31, *p* < 0.001) and confrontation (*F*_(4, 6120.9)_ = 2.92, *p* = 0.019) demonstrating that DT varied across trial conditions and confrontation types (Fig. 4). At last, higher environmental experience values predicted shorter DT (F_(1, 57.3)_ = 10.52, *p* = 0.002, β = −0.02 ± 0.007 s). The main effect of trial number was not significant.

In summary, DTs were longer in the migraine group, primarily due to extended DT in avoidance trials. Confrontation-related effects did not significantly differ between groups but influenced DT across groups in combination with specific trial conditions.

### Initial movement response (IMR)

Group differences in IMR were analyzed using a LMM as described in the Methods section, with environmental experience value (env_exp_val) as an additional covariate. A summary of all statistical calculations are provided [see Additional file 5].

Averaged across all conditions, the IMR value was substantially variable in both groups, with a numerically lower mean IMR in the migraine group (M = 227 ± 646) as compared to the control group (C = 291 ± 674).

**Fig. 5:**
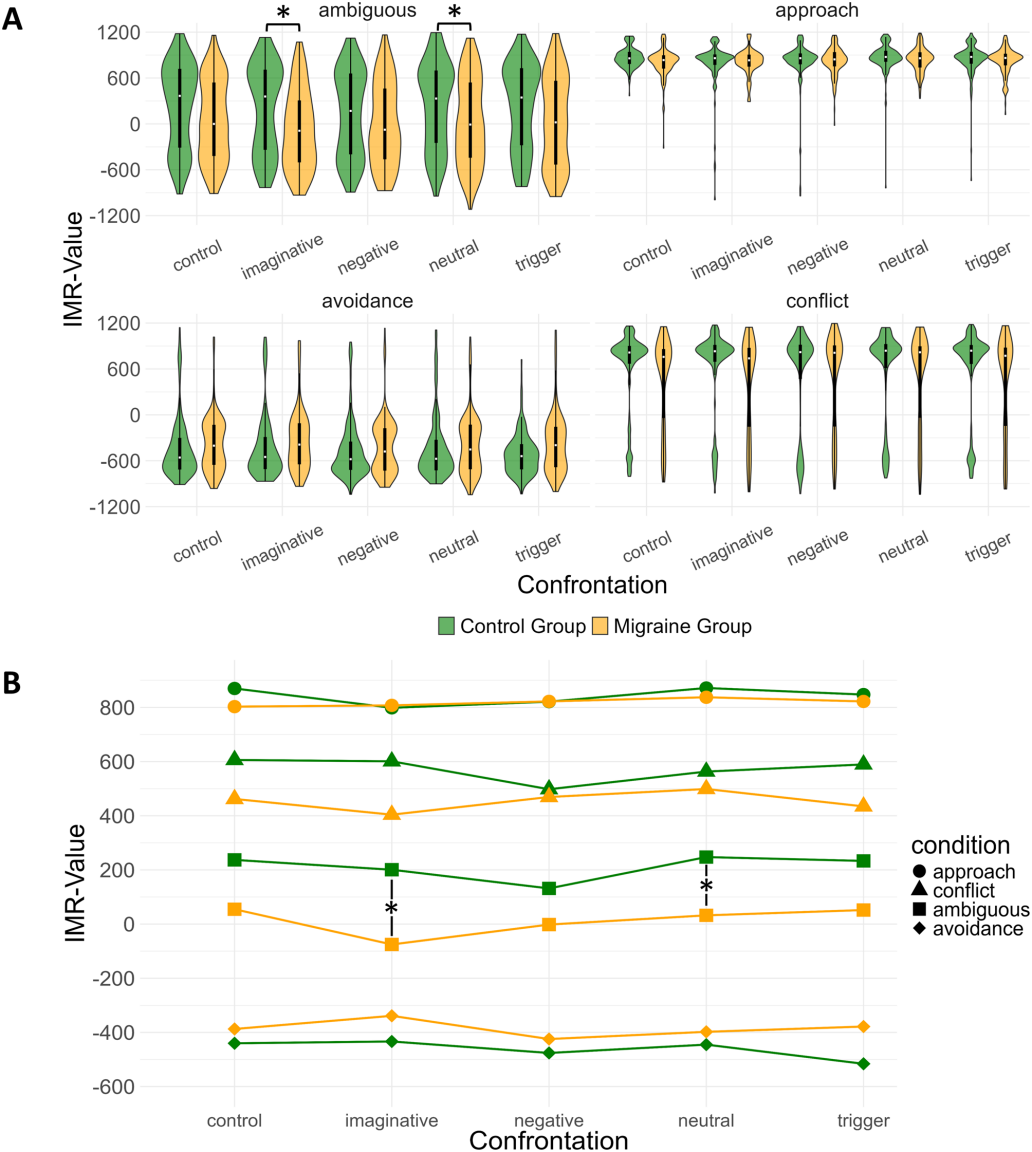
Initial movement response (IMR) values for groups, confrontation categories and trial conditions. **A**. Violin-Plot. **B**. Interactions-Plot. * = group differences with *p* < 0.05. For clarity, confrontation and condition-wise significance are not marked in this figure

The LMM found significant interactions. A three-way interaction between group, condition and confrontation was significant (F_(12, 6424.2)_ = 1.84, *p*=0.037), indicating an influence of the IMR by specific combinations of trial condition and confrontation categories that differ between both groups. Post-hoc tests revealed significantly lower IMR for the migraine group in ambiguous conditions following imaginative (β = -265.5 ± 105.0, *t*_(79.4)_ = -2.519, *p* = 0.013) and neutral confrontations (β = -206.0 ± 102.0, *t*(70.7) = -2.013, *p* = 0.048). Regarding the two-way interactions, only the combination of group x trial condition reached significance (F_(3, 58.2)_ = 4.08, *p* = 0.001). The main effect of trial condition was significant (F_(3, 58.2)_ = 374.79, *p* < 0.001), with predominantly positive IMR values in approach and conflict conditions, near-balanced responses in ambiguous conditions and predominantly negative IMR values in avoidance conditions (Fig. 5). Word confrontations also significantly affected the IMR (F_(4, 6424.1)_ = 4.66, *p* < 0.001). A significant effect of trial number indicated a gradual decrease in IMR across the experimental session (*F*_(1, 6476.8)_ = 241.62, *p* < 0.001, β = -1.87 ± 0.12). The main effect of group, on the other hand, reached no significance.

Taken together, these findings indicate that the lower IMR value of the migraine group does not emerge globally but is instead selective for specific combinations of trial condition and word confrontation, particularly under ambiguous conditions.

### EEG

#### Confrontation point

Before confrontation, participants exhibited low theta-band activity, accompanied by pronounced alpha-band activity and moderate power levels across higher frequency bands. The alpha-band activity was hereby more pronounced in the migraine group than in the control group.

**Fig. 6:**
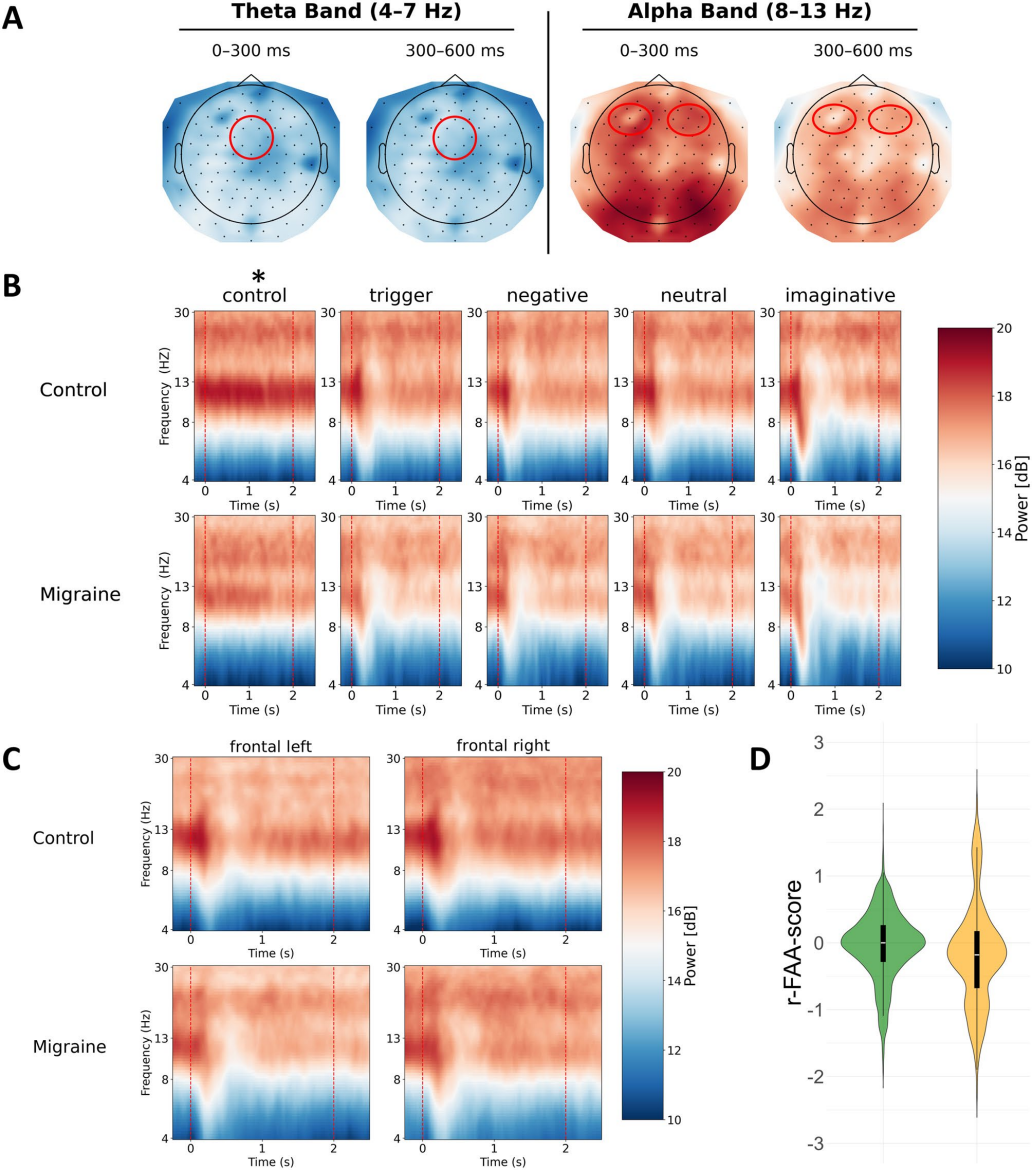
Time-Frequency-Analysis (TFR) at the confrontation phase that starts at 0s. **(A)** Topographic alpha and theta band power development over all participants. **(B)** TFR plots of FMT activity for the midfrontal cluster. **(C)** TFR plot of both frontal clusters over all trigger confrontation trials. **(D)** Violin Plot of the r-FAA-score across all confrontations. Red-lines mark either the region of interest or the time window used for the violin plot and statistical analysis. * = *p* < 0.05

Following the visual confrontations, but not the control confrontation – where no visual stimuli were shown – both groups demonstrated comparable power modulations within the midfrontal cluster (centered around FCz) as well as in the bilateral frontal clusters (around F3 and F4; Fig. 6). Within the midfrontal cluster, an increase in alpha-band power emerged immediately after the confrontation and persisted up to 100 ms post-confrontation. This was followed by a transient extension of the effect into the theta band approximately until 200 ms post-confrontation. Thereafter, theta power returned to pre-confrontation levels, whereas alpha power decreased below pre-confrontation levels, before returning to this level at approximately 1000 ms post-confrontation (Fig. 6A). A comparable temporal pattern was observed in the frontal clusters; however, here the pattern was largely confined to the alpha band, with a subsequent increase in theta band power (Fig. 6B).

The effects of group and confrontation on the FMT and FAA were analyzed using LMMs. Condition was not included as a fixed effect at this stage of the analysis, as the condition cue had not yet been presented. A full summary of all statistical calculations is provided [FAA: see Additional file 6, FMT: see Additional file 7].

For the FMT model, no significant interactions were observed. Instead, a significant main effect of confrontation (F_(3, 6545.0)_ = 5.95, *p* < 0.01) and of trial number (F_(1, 6545.1)_ = 275.99, *p* < 0.001) was found, indicating systematic changes in FMT across confrontation categories and over the course of the experimental session. The main effect of group did not reach significance. Post hoc comparisons indicated that the main effect of confrontation was driven by differences between all visual confrontation categories and the control condition (no visual presentation), whereas no significant differences were found among the visual confrontations.

The model assessing FAA values during the confrontation window revealed neither significant main effects nor interactions, indicating stable FAA values across confrontation categories, groups and time.

In summary, analyses within the confrontation window indicate that neither FMT nor FAA differed between the migraine and control groups. However, an increased power in the general alpha-band of the migraine group could be observed, and both FMT and FAA showed a specific modulation following visual confrontation.

#### Cue condition point

**Fig. 7:**
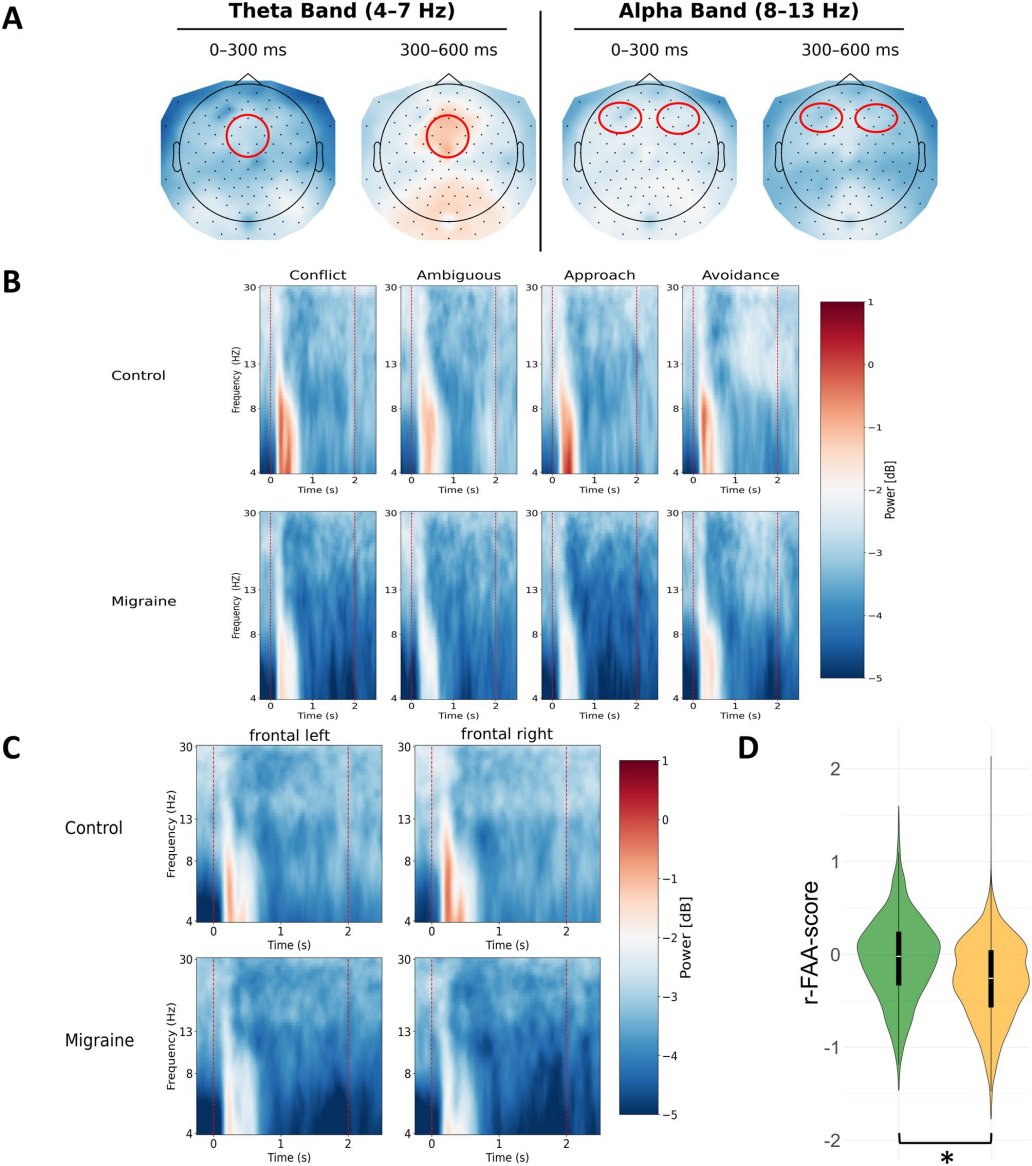
Time-Frequency-Analysis (TFR) averaged across trial conditions at the cue phase that starts at 0s. **(A)** Topographic alpha and theta band power development over all participants. **(B)** TFR plots of FMT activity for the midfrontal cluster. **(C)** TFR plot of r-FAA for approach conditions of both frontal clusters. **(D)** Violin Plot of the r-FAA-score averaged across all conditions. Red-lines mark either the cluster and region of interest (ROI) or the time window used for the violin plot and statistical analysis. * = *p* < 0.05

To examine all possible effects, a time-frequency analysis was performed for all trial conditions averaged across confrontations and for all confrontation conditions averaged across trial conditions (Fig. 7). Both showed a similar picture.

Before the condition cue was presented, participants exhibited low theta-band power, accompanied by higher power activity in the higher frequency bands. After the condition cue was presented, a transient increase in spectral power was observed in the first 500 ms of the decision phase at the midfrontal cluster and both frontal clusters. Power increases emerged approximately 100 ms after cue onset in alpha and theta band. Alpha-band power returned to pre-cue levels after approximately 100 ms, whereas theta-band power remained elevated until 500 ms post cue. Visually, the increase in oscillatory power was more pronounced in the control group than the migraine group.

The effects on FMT activity and FAA were analyzed using LMMs. A full summary of all statistical and significance calculations is provided [FAA: Additional file 8, FMT: Additional file 9].

For FMT activity, no significant interactions were significant. Yet, a significant main effect of group was observed (F_(1, 58.0)_ = 6.78, *p* = 0.012), indicating an overall difference in FMT between migraine and control group. In addition, a significant main effect of trial number was found (F_(1, 6536.1)_ = 8.82, *p* = 0.003), suggesting systematic changes in FMT over the course of the experimental session. All other main effects remained insignificant for the FMT activity.

For FAA values, the model revealed no significant interactions, while revealing significant main effects of group (F_(1, 58.0)_ = 6.88, *p* = 0.011), trial number (F_(1, 6535.3)_ = 19.3, *p* < 0.001) and condition (F_(3, 6535.0)_ = 3.42, *p* = 0.016). These results are indicating differences in FAA between groups, across experimental conditions, and over time. Post-hoc comparisons showed significantly higher right-side FAA scores following conflict trials compared to ambiguous condition trials (β = 0.029 ± 0.001, *t*_(6535.0)_ = 3.061, *p* = 0.012) over all participants.

Overall, the results suggest a difference between the migraine and control group in both FMT activity and FAA that is independent of task given variables, as well as condition-specific modulation of FAA in case of the ambiguous and conflict situations across all participants (see Fig. 7).

#### EEG-connectivity

During the confrontation window, dwPLI-based functional connectivity was examined as a function of group, confrontation condition, and cluster-link (i.e., inter-cluster connections). Separate LMMs were computed for the theta and alpha frequency bands. A summary of the statistical calculations with significant post-hoc tests is provided [see alpha: Additional file 10, theta: Additional file 11].

**Fig. 8:**
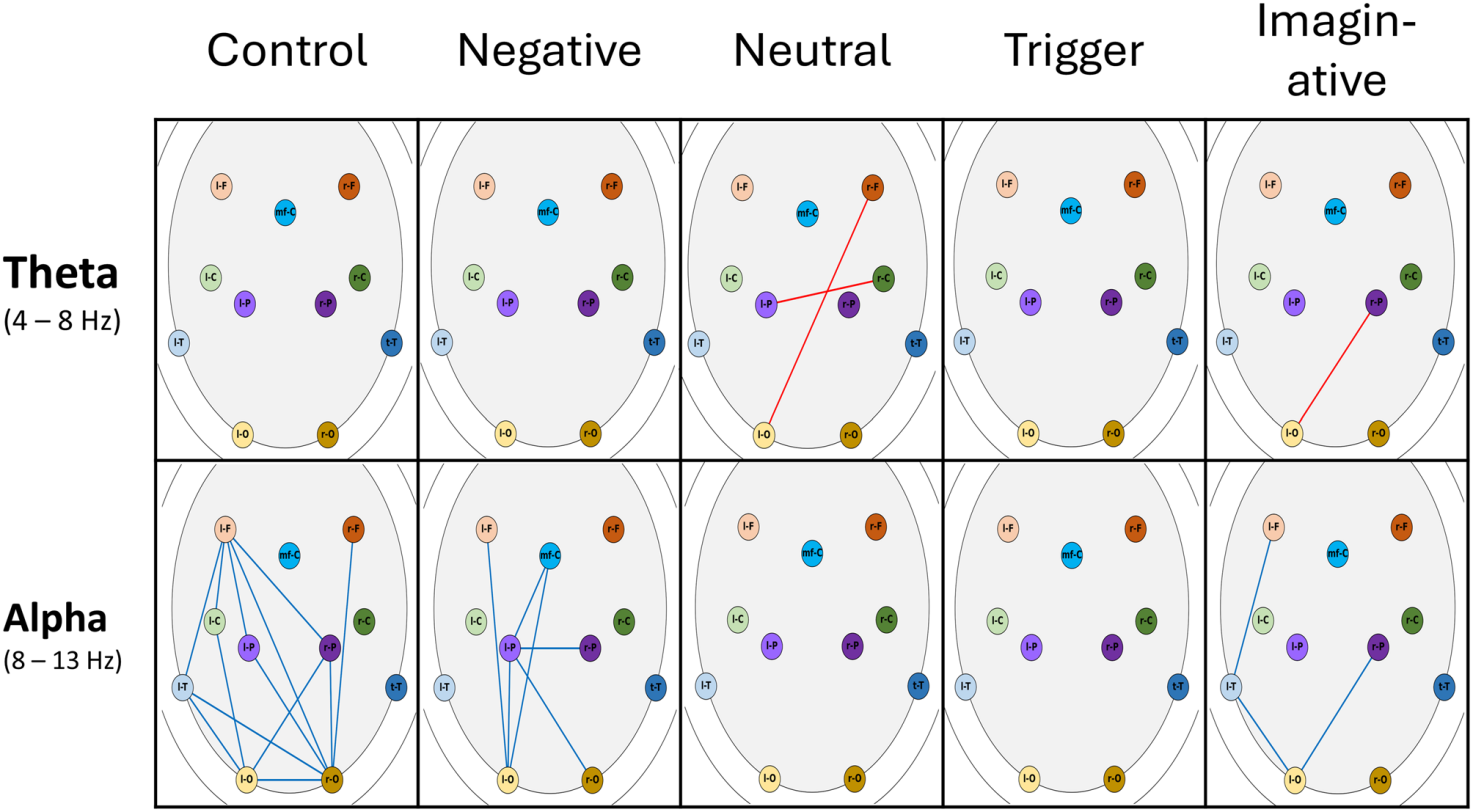
Difference in spatial dwPLI-based functional connectivity between different clusters of the migraine group as compared to the control group 2 s after confrontation. Red Lines = significantly higher dwPLI (*p* < 0.05). Blue Lines = significantly lower dwPLI-based functional connectivity (*p* < 0.05). For reasons of visual clarity, the locations of the clusters have been adjusted for this figure and clusters may not be placed at the center of their original location as shown in Fig. 1

For both frequency bands no significant three-way interaction was found. Two-way interactions reached significance for the interaction group x cluster-link (theta: F_(54, 15892)_ = 1.58, *p* = 0.004; alpha: F_(54, 15892)_ = 1.73, *p* < 0.001) and group x confrontation (theta: F_(4, 15892)_ = 7.79, *p* < 0.001; alpha: F_(4, 15892)_ = 20.21, *p* < 0.001) but for no other interaction. These interactions indicate that group-related differences in dwPLI were dependent on specific network connections and confrontation conditions rather than being uniformly expressed across the network. Post-hoc analyses revealed cluster-link-specific group differences in dwPLI (Fig. 8). In the theta band, a single connection showed increased dwPLI in the migraine group following imaginative confrontation, linking the left occipital and right parietal clusters. In contrast, the alpha band exhibited multiple cluster-links with reduced dwPLI in the migraine group. These effects occurred following negative, imaginative, and no-visual confrontations. They predominantly involved bilateral occipital connections as well as the left frontal cluster. For the main effects, significance was found for cluster-link (theta: F_(54, 15892)_ = 3.18, *p* < 0.001 | alpha: F_(54, 15892)_ = 3.19, *p* < 0.001) and confrontation (theta: F_(4, 15892)_ = 46.39, *p* < 0.001; alpha: F_(4, 15892)_ = 104.20, *p* < 0.001) but not for group.

Overall, these findings suggest that group differences in dwPLI-based functional connectivity during the confrontation window emerged primarily in the alpha band between specific network connections and task context rather than as global functional dwPLI-based connectivity alterations.

In the decision window, dwPLI-based functional connectivity was analyzed as a function of group, confrontation category, trial condition, and cluster-link (i.e., inter-cluster connections). Separate LMMs were computed for the theta and alpha frequency bands. A summary of the statistical calculations with significant post-hoc tests are provided [see alpha: Additional file 12, theta: Additional file 13].

**Fig. 9:**
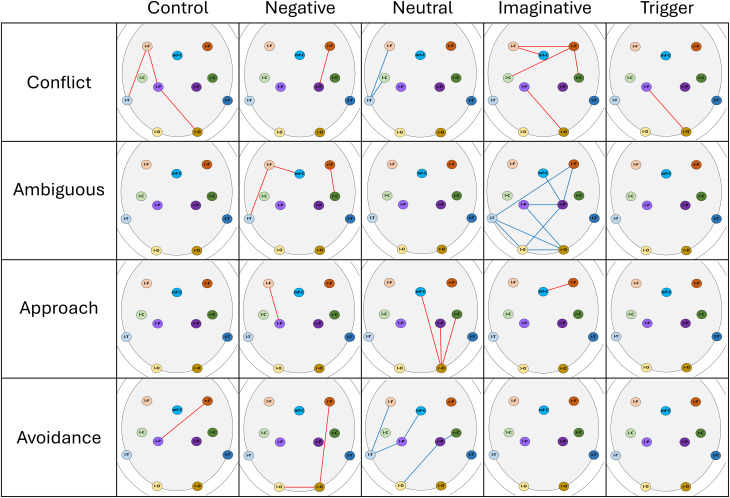
Difference in spatial theta band dwPLI-based functional connectivity between different clusters of the migraine group as compared to the control group across conditions. Red Lines = Significantly higher dwPLI (*p* < 0.05) Blue Lines = Significantly lower dwPLI (*p* < 0.05). For reasons of visual clarity, locations of the clusters have been adjusted for this figure and clusters may not be placed at the center of their original location as shown in Fig. 1

**Fig. 10:**
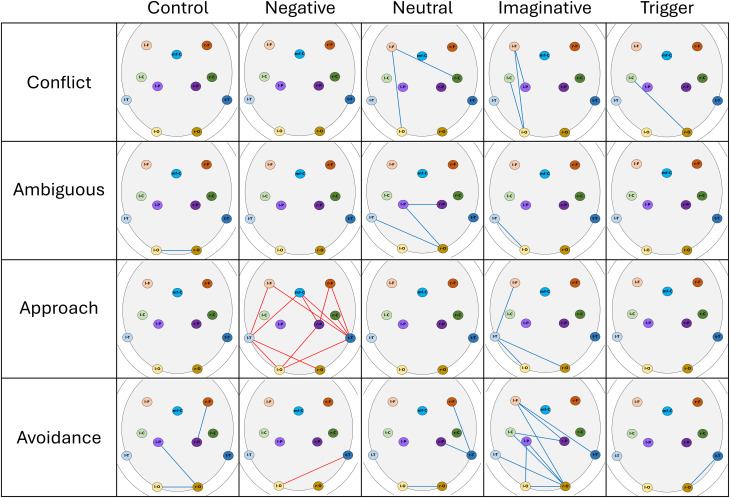
Difference in spatial alpha band dwPLI-based functional connectivity between different clusters of the migraine group as compared to the control group across conditions. Red Lines = Significant higher dwPLI (*p* < 0.05) Blue Lines = Significant lower dwPLI (*p* < 0.05). For reasons of visual clarity, locations of the clusters have been adjusted for this figure and clusters may not be placed at the center of their original location as shown in Fig. 1

The models found a significant three-way interaction of group x condition x trial condition (theta: F_(12, 63202.0)_ = 14.46, *p* < 0.001; alpha: F_(12, 63230.0)_ = 13.26, *p* < 0.001). Post-hoc analyses revealed group-related dwPLI differences that were specific to individual cluster links in the decision window (Figs. 9 and 10). Neither frequency band exhibited a consistent spatial dwPLI difference associated with a specific trial condition or confrontation category. Instead, dwPLI differences emerged for specific condition-confrontation combinations, each associated with either increased or decreased dwPLI across cluster-links.

In the theta band, the most pronounced group differences were observed in the avoidance condition and during negative- neutral and imaginative confrontations with differences involving occipital and frontal cluster-links. Increased dwPLI was observed exclusively during negative confrontation and only in combination with approach conditions, where effects notably involved the frontal midline cluster.

In the alpha band, group differences were primarily observed for frontal cluster-links across most condition-confrontation combinations. These effects were pronounced during negative confrontation, which was associated with increased dwPLI to the right frontal cluster in conflict and in most ambiguous and avoidance conditions. In contrast, the combinations conflict/neutral, avoidance/neutral and ambiguous/imaginative were associated with decreased dwPLI.

In both frequency-band models, trigger confrontation showed only minimal effects relative to other confrontation categories.

The models also found significant two-way interactions of condition x confrontation in both frequency bands (theta: F_(12, 63202.0)_ = 22.31, *p* = 0.001; alpha: F_(12, 63230.0)_ = 17.75, *p* < 0.001) but for no other interaction.

Regarding the main effects, significance was found for cluster-link (theta: F_(54, 63202.0)_ = 11.91, *p* < 0.001 | alpha: F_(54, 63230.0)_ = 2.90, *p* < 0.001), and confrontation (theta: F_(4, 63202.0)_ = 19.87, *p* < 0.001 | alpha: F_(4, 63230.0)_ = 10.86, *p* < 0.001). The main effect of trial condition was significant in the theta but not the alpha band. (theta: F_(3, 58.0)_ = 6.88, *p* < 0.001 | alpha: F_(3, 58.0)_ = 0.09, *p* = 0.964). The main effect group reached no significance in either frequency band model.

Taken together, these results suggest that group-related dwPLI-based functional connectivity differences were link-specific. They were dependent on particular combinations of confrontation categories and trial conditions, rather than reflecting a global alteration of functional network connectivity. Furthermore, whereas theta-band dwPLI was modulated by trial condition, alpha-band dwPLI was not. Across both frequency bands, no uniform pattern or functional connectivity differences emerged across conditions or confrontation categories.

### Post-hoc questionnaire

After the completion of the recording, the participants were asked about their subjective impression to several questions regarding the paradigm by rating on a scale between 0 (low value) and 10 (high value). Compared to the participants of the control group, the participants of the migraine group reported a higher subjective influence of the trigger word (C = 4 ± 2.3, M = 5.8 ± 2.5; t_(52)_ = 2.69, *p* = 0.01) and the imagination task (C = 4.5 ± 2.1, M = 5.9 ± 2.6; t_(52)_ = 2.16, *p* = 0.035) on their following approach and avoidance decision. The migraine group also reported that the whole session was more exhausting for them as compared to the report of the control group (C = 5.3 ± 2.5, M = 6.9 ± 1.5; t_(52)_ = 2.63, *p* = 0.011) and stated less personal motivation to gain as many points as possible (C = 8.9 ± 1.0, M = 7.7 ± 1.7; t_(52)_ = -3.11, *p* = 0.003). Finally, both groups acquired a similar score at the end of the paradigm, with no statistically significant difference (M = 384 ± 109, C = 427 ± 101; t_(54)_ = -1.43 s, *p* > 0.15).

### Exploratory subgroup analyses

Given the clinical heterogeneity of the migraine sample, exploratory subgroup analyses were performed comparing participants with episodic and chronic migraine. Only participants from the migraine group were included and the same LMM structure as described above was applied, with the factor group replaced by migraine diagnosis subtype. Subgroup assignment was based on participants' self-reported original clinical migraine diagnosis. Participants with chronic migraine who had improved under therapy remained assigned to the chronic migraine subgroup based on their previously established clinical diagnosis. As migraine subtype classification may also vary depending on current attack frequency, additional exploratory analyses using current migraine frequency-based subgroup assignment were considered to evaluate the robustness of the findings.

Participants with chronic migraine showed overall longer decision times (F(1, 24.55) = 4.70, *p* = 0.040) and altered behavioral values (F(1, 25.76) = 4.32, *p* = 0.048) compared to episodic migraine participants. No significant subgroup effects emerged for FAA or FMT during the confrontation window. During the cue condition window, a significant diagnosis × condition × confrontation interaction was observed for FMT activity (F(12, 3041.98) = 2.32, *p* = 0.006). Exploratory connectivity analyses additionally revealed significant diagnosis × confrontation interactions in both the alpha and theta frequency bands during the confrontation window. Using current migraine frequency-based subgroup assignment, several connectivity-related diagnosis interactions remained significant, particularly during the confrontation window, whereas behavioral and FMT-related subgroup effects were less consistent. However, no consistent subgroup-dependent EEG pattern emerged across analyses. Due to the limited subgroup sample sizes, these exploratory findings should be interpreted cautiously. Full statistical results for subgroup assignment based on previously established clinical diagnosis are provided in Additional file 14, while results for subgroup assignment based on current migraine attack frequency are provided in Additional file 15.

## Discussion

We designed this study to investigate whether indirect trigger confrontation modulates approach-avoidance decision-making and associated neurophysiological processes in individuals with migraine. Participants were exposed to visual stimuli and imaginative tasks prior to making approach and avoidance decisions within a virtual T-maze paradigm. The study addressed three main objectives: (I) validation of the approach-avoidance paradigm in the context of migraine-related trigger processing, (II) examination of group differences in behavioral responses (decision time and initial movement response), and (III) assessment of neurophysiological and connectivity-based differences, i.e. frontal alpha asymmetry [FAA], frontal midline theta [FMT], and dwPLI-based functional connectivity within predefined clusters.

Our data did not support the central hypothesis that indirect trigger confrontation would systematically modulate behavioral and neurophysiological outcomes, particularly by increasing avoidance-related responding in individuals with migraine. Trigger confrontation did not produce consistent effects either on behavioral performance or on EEG-based measures. Moreover, contrary to our initial hypothesis, individuals with migraine did not exhibit faster avoidance responses but instead showed overall prolonged decision times, particularly in avoidance-related conditions. The clearest findings were condition-specific effects in the T-maze task and overall behavioral and neurophysiological differences between participants with migraine and control participants. With regard to (I) paradigm validation, the task reliably elicited distinct condition-specific behavioral patterns in decision time and initial movement response across both groups, consistent with established models of conflict and uncertainty processing. However, condition-specific modulation of neurophysiological indices (FAA, FMT, and dwPLI-based connectivity) was limited and less consistent, suggesting only partial validation at the electrophysiological level.

In relation to (II) group differences in behavioral responses, individuals with migraine showed prolonged decision times and altered movement behavior, particularly in avoidance and conflict-related conditions, despite comparable overall task performance. These findings indicate increased cognitive load and altered decision dynamics in the migraine group.

Finally, addressing (III) neurophysiological and connectivity differences, individuals with migraine exhibited altered oscillatory activity and differences in functional connectivity, particularly involving frontal clusters and condition-dependent dwPLI patterns.

Beyond behavioral and psychophysiological measures, post-session assessments provided additional insight into the subjective experience of the task. Individuals with migraine reported increased exhaustion and a stronger perceived influence of trigger-related stimuli as compared to control participants.

### (I) validation of the T-maze paradigm

The trial conditions were explicitly designed to induce distinct behavioral and cognitive processes, and several converging findings indicate that this goal was largely achieved.

Behavioral results revealed condition-specific patterns across both groups. Decision times (DTs) were longest in combination with ambiguous and avoidance trials, followed by intermediate DTs in conflict trials and shortest DTs in approach trials (Table 2; additional file 4). This pattern is consistent with established models of decision-making under uncertainty and conflict, which predict an increased cognitive load under ambiguous or incongruent conditions, leading to prolonged decision times. Such effects are well described in the Decision Field Theory [31], the Diffusion Decision Model [32] and paradigms like the Stroop task [33].

Prolonged DTs in avoidance trials appear counterintuitive, as pure avoidance is neither inherently ambiguous nor conflictual. However, within the context of this paradigm – where participants were explicitly instructed to maximize reward – the avoidance condition likely constitutes a conflict situation, as it directly opposes the motivational goal. The virtual movement behavior of the participants, represented by the initial movement response (IMR), supports this interpretation. Positive IMR values, indicating movement toward the T-maze junction (approach), and negative values, indicating movement toward the safe room (avoidance), revealed distinct behavioral profiles for each trial condition in both groups (Fig. 5, Additional file 5).

Additional support for distinct approach-avoidance processing is provided by electrophysiological measures. Frontal alpha asymmetry (FAA) showed a pattern independent of the investigated paradigm variable, comparable to that reported in the foundational T-maze study [18], with increased r-FAA values in conflict as compared to ambiguous trial conditions. According to Gray’s revised reinforcement sensitivity theory (RST), higher r-FAA values are associated with approach behavior, whereas lower values reflect either withdrawal or uncertainty and conflict resolution [11]. In line with this theory, conflict trials exhibited lower r-FAA values, faster DTs and increased approach behavior as compared to ambiguous trials.

However, mean FAA values across the remaining conditions remained largely uniform and did not display the pronounced condition-specific differences expected and reported before in this context [18]. Similarly, frontal midline theta (FMT) activity – commonly associated with conflict monitoring and cognitive control – did not differ significantly between trial conditions.

Several explanations may account for the absence of robust FAA and FMT differences. Firstly, FMT activity is highly dependent on the specific decision task employed [12], and the T-maze paradigm itself or its adaptations were originally designed to investigate FAA and not optimized to elicit condition-specific theta modulation. For example, the required conflict in this paradigm is continuous and also changing over time by requiring constant movement toward or away from a specific place in contrast to typical FMT optimized paradigms that only require a single punctual one-time decision [34]. Secondly, the substantial interindividual variance in DTs may have resulted in imprecise time-locking of neurophysiological activity to the decision process, thereby “smoothing” condition-specific effects in the grand average.

Nevertheless, temporal alignment between behavioral and electrophysiological measures suggests the interpretation that FAA and FMT captured late-stage decision processes. Mean frontal and midfrontal activity terminated approximately 700 ms after cue onset, aligning with mean DTs of 900-1000 ms when accounting for approximately 200 ms delay between conscious decision formation and movement initiation [35]. This temporal correspondence suggests that the recorded neural activity reflects processes involved in the final stages of decision-making.

As an additional level of approach-avoidance behavior, the indirect confrontation was introduced in the form of visual stimuli and imaginative tasks immediately before the approach-avoidance decision phase. While there is evidence that indirect confrontation influenced certain measures, these effects were limited to specific confrontation-condition combinations. The individually tailored trigger confrontations did not produce measurable effects beyond post-session self-reports. Although FMT activities are known to be particularly sensitive to confrontation effects, significant differences were only observed between the control and the other confrontation categories. Functional connectivity analyses further revealed confrontation-specific changes in dwPLI (e.g. negative, imaginative and neutral confrontation), although we expected possible differences in functional connectivity to be visible over all trial conditions [13]. Instead, we found unique patterns for specific confrontation and trial conditions combinations.

As with the FAA and FMT findings, these limited effects may reflect insufficient time-locking or indicate that indirect confrontation is not strong enough to consistently manipulate approach-avoidance responses within this paradigm.

In summary, the adapted T-Maze paradigm reliably elicits behavioral and neural markers of approach-avoidance processing although with certain limitations. Additional modulation of these responses with indirect confrontation, remained inconsistent. Future studies employing reduced task complexity and more precise temporal alignment are required to determine whether these limitations arise from methodological constraints or from the paradigm design itself.

### (II + III) differences between migraine and control group in behavioral and neurophysiological responses

Individuals with migraine exhibited alterations in approach-avoidance decision-making characterized by increased cognitive load and reduced top-down regulatory control.

This is supported by the consistently prolonged decision times (DTs) elicited within our T-maze task. Previous studies have shown that increased DTs in approach-avoidance paradigms are indicative of elevated cognitive load and heightened decision conflict [36].

At the neurophysiological level, this behavioral finding is paralleled by altered oscillatory activity after the confrontation and during the decision-making phase (Figs. 6 and 7). Compared to migraine free individuals, those with migraine exhibited overall reduced alpha-band power. In the frontal areas of the cortex, higher frontal alpha power – as observed in the control group – is consistent with the Active Inhibition Hypothesis, reflecting efficient top-down regulation and inhibitory control [37]. In contrast, reduced alpha power – as observed in the migraine group – suggests more desynchronized cortical processing, indicative of increased neural activity. When combined with reduced theta power (e.g. in the decision-making phase) and longer decision times, these findings point toward less efficient recruitment of prefrontal control and conflict-monitoring processes.

These results align with previous literature describing increased cortical responsiveness and reduced regulatory capacity in individuals with migraine, leading to heightened sensitivity to external stimuli [13]. This interpretation is further supported by previous evidence of impaired multisensory integration and altered cognitive processing in migraine, particularly in chronic migraine populations [38]. Such altered neural processing may also account for the increased subjective exhaustion reported by the migraine group following the experimental session.

At a broader network level, previous neuroimaging studies have described altered prefrontal and brainstem connectivity in migraine, consistent with impaired cognitive and sensory regulatory mechanisms [39, 41]. In line with this, the present functional connectivity analyses revealed decreased dwPLI-based connectivity involving frontal electrode clusters across multiple condition-confrontation combinations.

We therefore interpret prolonged DTs as the behavioral expression of reduced regulatory control, with oscillatory and connectivity findings providing converging neurophysiological evidence.

Increased exhaustion or reduced motivation may also have contributed to prolonged decision times. However, the absence of group differences in the approach condition argues against a purely generalized slowing effect and instead suggests condition-specific alterations in behavioral responding.

Further insight is gained by examining DT differences across trial conditions. The largest group differences were observed in avoidance trials, followed by ambiguous and conflict trial conditions, whereas no indications for differences emerged in approach trial conditions. This pattern was unexpected, as we initially hypothesized that individuals with migraine would exhibit faster avoidance responses. Instead, migraine participants required more time to reach a decision.

This raises the question of whether prolonged DTs are primarily driven by conflict intensity or by the degree of avoidance inherent in each trial condition. Notably, the only trial condition without a group difference – the approach condition – lacked both components. Although avoidance trials, as previously noted, could be interpreted as involving strong approach-avoidance conflict due to opposing motivational cues, previous findings indicate that clearly avoidance related trial conditions typically elicit shorter DTs than ambiguous ones. This observation favors the interpretation that increased conflict, rather than avoidance alone, underlies the prolonged DTs observed in the migraine group.

Finally, frequency-domain analyses revealed lower r-FAA scores and reduced FMT activity in individuals with migraine, independent of trial condition. According to Gray’s RST, this can be associated with enhanced conflict sensitivity and withdrawal tendencies, whereas reduced FMT activity is typically linked to lower levels of decision conflict requiring less cognitive control. This apparent discrepancy may indicate that the present paradigm did not reliably elicit canonical FMT responses associated with conflict monitoring or was highly cognitively demanding and is therefore eliciting an FMT increase across all conditions and confrontations.

### The role of the indirect trigger confrontation

Contradicting the original hypothesis, we found that trigger confrontation did not systematically influence decision-making in individuals with migraine and did not elicit robust differences as compared to migraine-free control individuals.

These results contrast the subjective reports of individuals with migraine, who indicated that the trigger words strongly influenced their decisions. However, this perceived influence is not reflected by objective behavioral or neurophysiological measures. Specifically, no significant effects of trigger confrontation were observed on DTs or IMR.

At the level of cognitive processing, trigger confrontation did not induce measurable changes, either immediately following the confrontation or during the subsequent decision-making phase. Similarly, functional connectivity analysis revealed only minimal dwPLI differences between groups, following indirect trigger exposure. This was observed despite the fact that certain confrontation categories, such as the negative confrontation, were able to increase the wide network dwPLI-based functional connectivity in the theta band to some extent.

With respect to imaginative trigger confrontations, small effects were detectable in combination with specific trial conditions; however, these effects remained subtle and were primarily reflected in group differences in dwPLI rather than consistent behavioral or oscillatory changes.

Taken together, these findings suggest that the indirect trigger confrontation primarily activated migraine-related cognitive associations without consistently modulating behavioral or neurophysiological responses. Importantly, confrontation was limited to semantic processing of trigger-related words and therefore may not sufficiently engage the multisensory and interoceptive pathways involved in real-world migraine trigger exposure. This may partly explain why confrontation-related effects emerged only in highly specific condition-confrontation combinations rather than as robust generalized effects.

The above outlined interpretation is dependent on the assumption that both groups started the experiment in a comparable mental state. However, this assumption may not hold. Migraine participants could have already been in a heightened reactive state due to their expectations about the study or because they rated the trigger words at the beginning. Such a “pre-triggered” state could have persisted throughout the experiment, potentially explaining the overall group differences, the increased cognitive load and exhaustion, and the lack of observable effects of the trigger confrontation. Additionally, such an anticipatory state, combined with the sustained cognitive demand of the T-maze task, may have produced ceiling effects in cognitive or affective processing measures.

Another possible explanation is that the observed differences in the migraine group are driven by an unidentified subgroup. For example, participants with exceptionally long decision times could increase the variance in the patient group and dilute EEG signals due to poor time-locking. This might account for the absence of significant group x condition interactions in FMT or FAA, as well as the lack of a clear pattern in functional connectivity. One contributing factor may be migraine-related heterogeneity, including differences in migraine phase at the time of testing. Such variability could have increased between-subject variance and reduced temporal alignment of EEG responses, thereby obscuring condition-specific effects. In addition, the continuous and dynamically evolving conflict structure of the T-maze differs from paradigms classically optimized for FMT elicitation, which typically rely on discrete and temporally well-defined conflict events. This may have reduced the sensitivity of FMT as a marker of conflict monitoring in the present design. The high task complexity, combined with preceding trigger confrontations, may have obscured potential effects.

## Limitations and future directions

A key limitation of the present study is the fact that not all participants were in a strict interictal state. About 40% of our participants were tested in close temporal proximity to migraine attacks, particularly among chronic migraine patients. State-dependent fluctuations across the migraine cycle may therefore have increased between-subject variance while reducing sensitivity for detecting robust behavioral and neurophysiological effects. At the same time, restricting participation strictly to a predefined interictal interval would have limited the ecological validity of a paradigm investigating trigger-related processing and dynamic symptom sensitivity, particularly in chronic migraine where prolonged attack-free intervals are often difficult to achieve.

In addition, the migraine sample was clinically heterogeneous, including episodic and chronic migraine as well as variability in aura status and disease characteristics, which may have further increased between-subject variance and masked subtle condition-specific neurophysiological effects. Exploratory subgroup analyses revealed selective behavioral and EEG-connectivity differences in chronic migraine; however, these findings were inconsistent across measures and should be interpreted cautiously. These exploratory findings further suggest that migraine subtype may contribute to variability in behavioral and connectivity-related measures, although the present sample size was insufficient for reliable subgroup characterization.

Further limitations relate to the experimental design itself. The indirect trigger confrontation relied primarily on semantic processing of trigger-related words and may therefore not adequately reflect the multisensory and interoceptive nature of real-world migraine triggers. Moreover, temporal variability in decision latency, the continuous nature of the T-maze paradigm, and overall task complexity may have reduced the sensitivity of FAA, FMT, and connectivity measures to detect subtle condition-specific effects. Finally, the present study may have been underpowered to detect subtle interaction effects between group, condition, and confrontation. Future studies should therefore incorporate prospective headache diaries, standardized migraine phase assessment, and larger clinically stratified samples together with simpler and more temporally discrete paradigms.

## Conclusions

The here introduced T-Maze paradigm was suitable to investigate behavior in approach-avoidance situations. Individuals with migraine exhibited altered behavioral and neurophysiological responses during approach-avoidance decision-making, potentially reflecting increased cognitive load and reduced top-down regulatory control. Indirect trigger confrontation did not increase avoidance behavior in individuals with migraine. Although it may have influenced brain processing, its effect on decision-making remains unclear. Instead, robust differences between individuals with and without migraine were observed, particularly in conflict, ambiguous and avoidance decisions. However, it could not be determined whether these reflect baseline differences in mental state. Future studies may employ simpler behavioral paradigms, collect more detailed patient information, and consider basic tasks such as the emotional Stroop-task to better assess indirect trigger effects. Additionally, neuromodulation targeting FAA or FMT could be explored to investigate their causal role in the observed group differences.

## Supplementary Information

Below is the link to the electronic supplementary material.

Supplementary Material 1: Visual Trigger Confrontation Library – German Version

Supplementary Material 2: Retrospective assessment of migraine-attack proximity at the time of testing. Footnote: Retrospective information regarding proximity to migraine attacks was obtained after data collection through patients’ migraine diaries, where available. Two participants developed migraine symptoms during testing and were therefore excluded from the final analyses and are not listed in this table

Supplementary Material 3: Characteristics of the study population (*n* = 60). ^1^Among these, 5 reported a recent reduction in migraine frequency under medication

Supplementary Material 4: Summarized Statistical Table - Decision Time (DT). Significant effects (*p* < 0.05) are highlighted in grey

Supplementary Material 5: Summarized Statistical Table - Initial Movement Response (IMR). Significant effects (*p* < 0.05) are highlighted in grey

Supplementary Material 6: Summarized Statistical Table - FAA after Confrontation. Significant effects (*p* < 0.05) are highlighted in grey

Supplementary Material 7: Summarized Statistical Table - FMT after Confrontation. Significant effects (*p* < 0.05) are highlighted in grey

Supplementary Material 8: Summarized Statistical Table - FAA in Decision-Phase. Significant effects (*p* < 0.05) are highlighted in grey

Supplementary Material 9: Summarized Statistical Table - FMT in Decision-Phase. Significant effects (*p* < 0.05) are highlighted in grey

Supplementary Material 10: Summarized Statistical Table - Cluster-Link Alpha Band dwPLI-based functional connectivity after Confrontation. Significant effects (*p* < 0.05) are highlighted in grey

Supplementary Material 11: Summarized Statistical Table - Cluster-Link Theta-Band dwPLI-based functional connectivity after Confrontation. Significant effects (*p* < 0.05) are highlighted in grey

Supplementary Material 12: Summarized Statistical Table - Cluster-Link Alpha-Band dwPLI-based functional connectivity in the Decision Phase. Significant effects (*p* < 0.05) are highlighted in grey

Supplementary Material 13: Summarized Statistical Table - Cluster-Link Theta-Band dwPLI-based functional connectivity in the Decision Phase. Significant effects (*p* < 0.05) are highlighted in grey

Supplementary Material 14: Exploratory subgroup analyses comparing participants with episodic and chronic migraine based on the self-reported original migraine diagnosis. Exploratory subgroup analyses were conducted using linear mixed-effects models comparing participants with episodic and chronic migraine. Only statistically relevant effects are shown for clarity. Non-significant effects and interaction terms are omitted

Supplementary Material 15: Exploratory subgroup analyses comparing participants with episodic and chronic migraine based on the self-reported headache days per month (chronic ≥ 15 headache days). Exploratory subgroup analyses were conducted using linear mixed-effects models comparing participants with episodic and chronic migraine. Only statistically relevant effects are shown for clarity. Non-significant effects and interaction terms are omitted

## Data Availability

The datasets used and/or analyzed during the current study are available from the corresponding author on reasonable request.

## Abbreviations

DT: Decision-Time
dwPLI: debiased weighed Phase-Lag-Index
FAA: Frontal Alpha Asymmetry
FMT: Frontal-Midline-Theta
IMR: Initial Movement Response
LMM: Linear Mixed Effect Modell
r-FAA: right Frontal-Alpha-Asymmetry
