# Supplementary figures and images for: Migraine is associated with altered approach–avoidance decision processing: a case control study
Source: J Headache Pain. 2026 Jul 16;27(1):183. doi: 10.1186/s10194-026-02445-5 (PMC13377724; doi:10.1186/s10194-026-02445-5)

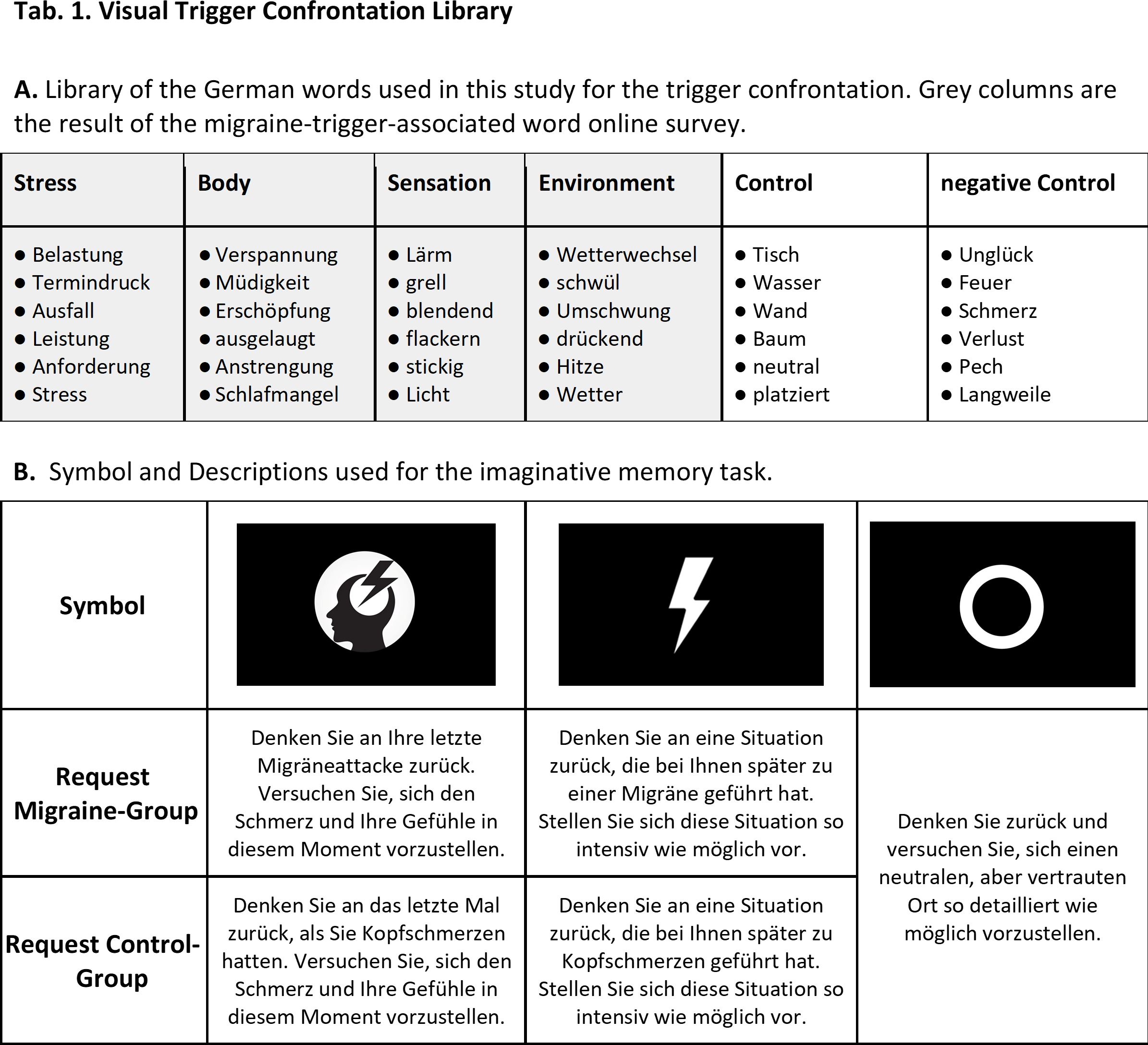

Supplement: Supplementary file 1 — Supplementary Material 1: Visual Trigger Confrontation Library – German Version [file 10194_2026_2445_MOESM1_ESM.tif]
